# Dual N- and C-Terminal Helices Are Required for Endoplasmic Reticulum and Lipid Droplet Association of Alcohol Acetyltransferases in *Saccharomyces cerevisiae*

**DOI:** 10.1371/journal.pone.0104141

**Published:** 2014-08-05

**Authors:** Jyun-Liang Lin, Ian Wheeldon

**Affiliations:** Department of Chemical and Environmental Engineering, University of California Riverside, Riverside, California, United States of America; Simon Fraser University, Canada

## Abstract

In the yeast *Saccharomyces cerevisiae* two alcohol acetyltransferases (AATases), Atf1 and Atf2, condense short chain alcohols with acetyl-CoA to produce volatile acetate esters. Such esters are, in large part, responsible for the distinctive flavors and aromas of fermented beverages including beer, wine, and sake. Atf1 and Atf2 localize to the endoplasmic reticulum (ER) and Atf1 is known to localize to lipid droplets (LDs). The mechanism and function of these localizations are unknown. Here, we investigate potential mechanisms of Atf1 and Atf2 membrane association. Segments of the N- and C-terminal domains of Atf1 (residues 24–41 and 508–525, respectively) are predicted to be amphipathic helices. Truncations of these helices revealed that the terminal domains are essential for ER and LD association. Moreover, mutations of the basic or hydrophobic residues in the N-terminal helix and hydrophobic residues in the C-terminal helix disrupted ER association and subsequent sorting from the ER to LDs. Similar amphipathic helices are found at both ends of Atf2, enabling ER and LD association. As was the case with Atf1, mutations to the N- and C-terminal helices of Atf2 prevented membrane association. Sequence comparison of the AATases from *Saccharomyces, non-Saccharomyces* yeast (*K. lactis* and *P. anomala*) and fruits species (*C. melo* and *S. lycopersicum*) showed that only AATases from *Saccharomyces* evolved terminal amphipathic helices. Heterologous expression of these orthologs in *S. cerevisiae* revealed that the absence of terminal amphipathic helices eliminates LD association. Combined, the results of this study suggest a common mechanism of membrane association for AATases via dual N- and C-terminal amphipathic helices.

## Introduction

Alcohol acetyltransferase (AATase; E.C. 2.3.1.84) produces acetate esters through the condensation of an alcohol and acetyl-coenzyme A (CoA). Owing in large part to the aromas and flavors of volatile aliphatic and alicyclic esters the biochemistry of AATase in wine and brewer’s yeasts has been extensively studied [1]–[4]. For example, during fermentations 2-phenyl ethanol and CoA condense to phenyl ethyl acetate to produce a flowery aroma reminiscent of roses. Similarly, synthesized ethyl and isoamyl acetates produce scents of sweet pear and banana, respectively. In *Saccharomyces cerevisiae* these reactions are catalyzed by two AATases, Atf1 and Atf2 [5], [6]. Double knockouts of these enzymes eliminated isoamyl ester synthesis and reduced ethyl acetate synthesis by 50% [7]. Single knockouts of the two homologs demonstrated that Atf1 is primarily responsible for acetate ester synthesis, but broad substrate specificities lead to the production of a range of linear and branched esters that add to the complexity of aromas during fermentations [7]–[9]. With respect to expression, it has been shown that the transcription of *ATF1* is negatively regulated by oxygen and unsaturated fatty acids [10], [11] and that fermentations conditions (temperature, nitrogen content and glucose concentration) can significantly alter Atf activity and the resulting volatile ester profiles [12], [13].

While the biochemistry of AATases in *S. cerevisiae* is well studied, comparatively little is known about their structure. There are no crystal structures of Atf from *Saccharomyces* or *non-Saccharomyces* yeast and there are no suitable templates to generate high confidence homology models. Atf1 from *S. cerevisiae* and large Atf1 (lgAtf1) from *S. pastorianus* have high sequence similarly (83% similarity, 77.5% identity), but there is little sequence similarity between Atf1 and Atf2 (48.9% similarity, 34.5% identity). Despite a conserved H-X-X-X-D putative active site, there is even less similarly between Atf1 from *S. cerevisiae* and from Atfs from *non-Saccharomyces* yeast and various fruit species [14]–[16].

It is known that Atf1 localizes to the endoplasmic reticulum (ER) and to lipid droplets (LDs). Early biochemical studies isolated Atf activity from microsomal cell fractions [4], [17] and C-terminal GFP-tagged Atf1 confirmed LD localization by Nile Red co-staining [18]. High throughput screening suggests ER localization of both Atf1 and −2 [19]. Atf2 activity has been isolated in cell fractions that possibly contained LDs and in cell membrane fractions [20]. In addition, fluorescence imaging of GFP-tagged Atf2 and fractionation with the ER marker Wbp1 are evidence of ER localization [21].

LDs are dynamic organelles that serve as neutral lipid storage depots and hubs for lipid metabolism [22]. Across eukaryotes, evidence suggests that the monolayer phospholipid membrane of LDs and their neutral lipid contents emanate from the ER [23], [24]. In *S. cerevisiae*, LDs are composed of near equal molar ratios of triacylglycerols (TAGs) and sterol esters (SE) and proteomic studies reveal that upwards of 40 different proteins can localize to LDs, many of which also localize to the ER [25]–[27]. A number of LD association mechanisms are known [23]: amphipathic helices can directly bind to the monolayer LD membrane; terminal hydrophobic domains can embed into LDs; and, internal hydrophobic hairpin loops can anchor to the LD membrane. It is also possible that post-translation lipidation provides a lipid anchor and that LD associated proteins provide protein-protein interactions for anchoring. For many LD proteins the mechanism of targeting remains an open question. LD proteins such as perilipins are known to traffic from the cytosol to LD and associate by hairpin loops; however, the mechanisms of sorting proteins from the ER to LD have yet to be determined [28], [29].

While there is strong evidence for Atf1 localization to the ER and LDs, the mechanism of this association remains unclear. Atf2 is also membrane-associated and has been shown to localize to the ER; however, the extent of this localization and the possibility of sorting to LDs in a similar manner as Atf1 has not been explored. There are no obvious lipidation sequences within Atf1 or −2, and there are no predicted transmembrane domains [30], [31]. Helical domains at the N- and C-termini of both Atf1 and −2 that we predicted to be amphipathic in nature suggest possible membrane-association domains and are the origin of our hypothesis: the N- and/or C-terminal helical domains of Atf1 and −2 may be required for membrane association. Here, we use a series of N- and C-terminal deletions and selected point mutations to test our hypothesis. We demonstrate that the predicted N- and C-terminal AHs of Atf1 are necessary for LD localization and that membrane association originates at the ER. In addition, we discovered that Atf2 also localizes to LDs, and that the conserved helical structures of Atf2 are also essential for ER and LD localization. The absence of such evolved structures in *non-Saccharomyces* yeast and certain fruit disables membrane association to the ER and LDs in *S. cerevisiae*.

## Material and Methods

### Strains and culture conditions

The *S. cerevisiae* strain BY4742 was used in all experiment of this study. Cells harvesting expression plasmids were grown in synthetic minimal (SD) medium containing 0.67% yeast nitrogen base (Becton-Dickinson), amino acid supplements (Sunrise), and 2% glucose. To promote LD synthesis, cells were cultured in oleic acid medium containing 0.67% yeast nitrogen base, 0.1% yeast extract, 0.2% Tween 40, and 0.1% oleic acid according to [32]. Cells were first cultured in SD medium to late log phase prior to washing and subsequent culturing in oleic acid medium for an additional 48 h.

### Plasmids

An overexpression cassette with C-terminal GFP tag driven by the constitutive yeast promoter phosphoglycerate kinase 1 (PGK1p) and terminated by PGK1t was constructed. The promoter and terminator were amplified from genomic DNA of BY4742 and cloned into pRS426. PGK1p was inserted using SacI and SacII sites. PGK1t was inserted using SpeI and KpnI sites, while GFP was inserted at NotI and SpeI sites. All *ATF* genes were cloned into PGK1 overexpressing cassette using SacII and NotI sites. *ATF1*, *ATF2* and truncations to *ATF1* were amplified from BY4742 genomic DNA. *ATF1* and −*2* variants with point mutations were designed and synthesized by a gene assembly method [33]. Briefly, the N-terminal 195 base pairs (bp) and C-terminal 201 bp gene fragments with mutations were assembled and fused to the rest gene using overlap extension PCR. *ATF* genes from *S. pastorianus* (AY242066), *S. lycopersicum* (NP_001234496), and *C. melo* (BAB78588) were also assembled by gene assembly and codon optimized for yeast expression. *ATF*s from *K. lactis* (XM_455762), and *P. anomala* (JN701429) were amplified from genomic DNA. pERGmDsRed (a gift from J. Goodman, University of Texas Southwestern Medical School) was used to express an LD marker Erg6 [34]. A cassette consisting DsRed-PGK1t-LEU2 for C-terminal tagging of chromosomal genes was constructed by sequential cloning. DsRed, amplified from pERGmDsRed, was first inserted into pRS426 containing PGK1p-PGK1t at NotI and SpeI sites. LEU2, amplified from pRS315, was subsequently inserted between KpnI and EcoRV sites. This cassette was used to fuse DsRed to the 3′ end of the ER marker Sec61 and the LD marker Erg6 by PCR-based homologous recombination as previously described [35].

### Oil red staining

Cells grown to stationary phase were washed twice with phosphate buffer saline (PBS), fixed with 3.7% formaldehyde for 45 min, and washed twice more with PBS. Fixed cells were stained with Oil Red O solution (Sigma) for 2 min and washed twice with PBS before fluorescent microscopy imaging. Oil Red O solution was prepared by diluting the stock solution with two volumes of deionized water and filtered with a 0.45 µm syringe filter.

### Fluorescent microscopy and quantification of LD/ER localization

Cells were observed with an Olympus BX51 microscope (UPlanFL 100X 1.30 oil-immersion objective lens, mercury lamp) and fluorescent images were captured by a Q-Imaging Retiga Exi CCD camera. Images were processed using CellSens Dimension 1.7 software (Olympus). The quantification of LD and ER localization was accomplished by evaluating a total of 300 or more cells from three separate experiments. LD localization was considered positive if a given cell with nascent, premature, or mature LDs exhibited GFP fluorescence around the circumference of the LD. ER localization was considered positive if GFP fluorescence appeared in a pattern matching that of the Sec61 ER marker.

### Subcellular fractionation

LD isolation was carried out as previously described [36] with slight modifications. Cells were cultivated to stationary phase, harvested, and transformed into spheroplasts by zymolyase 20 T (Seikagaku Corporation, Japan). Spheroplasts were resuspended in LD-A buffer (12% Ficoll, 10 mM MES/Tris pH 6.9, 0.2 mM EDTA, 1 mM PMSF) and homogenized with 30 strokes in a Doune Homogenizer (Sartorius). Cell debris was removed by centrifugation at 6000 rpm for 10 min using a Sorvall SS34 rotor. The collected homogenate was overlaid with buffer LD-A in an Ultra-Clear centrifuge tube (Beckman) and centrifuged at 25,000 rpm for 1 h using a SW-28 swing bucket rotor (Beckman). The supernatant was saved as the cytosolic fraction, and the floating white layer on top (the crude LD fraction) was collected and homogenized gently with 7 strokes in a Dounce Homogenizer. The resulting homogenate was overlaid with buffer LD-B (8% Ficoll, 10 mM MES/Tris pH 6.9, 0.2 mM EDTA, 1 mM PMSF) in a new centrifuge tube and centrifuged at 25,000 rpm for 30 min. The resulting white layer on top was used as the purified LD fraction. Five-µg, determined by Bradford reagent (Sigma), of each subcellular fractionation sample was resolved by SDS-PAGE and western blot analysis. To detect proteins, the following antibodies were used: rabbit anti-GFP (Invitrogen), goat anti-rabbit IgG-HRP (Invitrogen), goat anti-DsRed (Santa Cruz Biotech), donkey anti-goat IgG-HRP (Santa Cruz), mouse anti-β actin (mouse), and goat anti-mouse IgG-HRP (Thermo Fisher Scientific).

## Results

### N- and C-terminal domains are required for membrane association of Atf1

**Figure 1:**
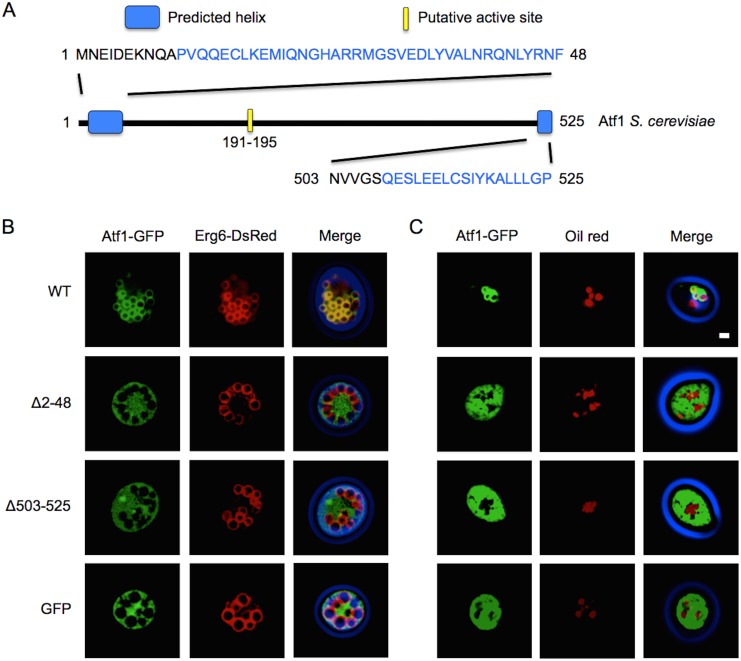
The N- and C-terminal domains of Atf1 are necessary for lipid droplet localization. (A) Atf1 from *S. cerevisiae* with the location of the putative active site and predicted N- and C-terminal helices. The amino acid sequence of the terminal helices are shown. (B) Fluorescent microscopy images of selected stationary phase cells cultured in oleic acid media co-expressing Atf1-GFP and the LD marker Erg6-DsRed (Ex. 400±50, Em. 500±25). Atf1-GFP signal is shown in green and Erg6-DsRed is shown in red. The merged image shows the phase contrast image false colored in blue. Co-localization of green and red is indicated by yellow and demonstrates Atf1-GFP localization to LDs in the wild type (WT) Atf1. A GFP control shows cytosolic localization. (C) Fluorescent images of stationary phase cells cultured in glucose media overexpressing Atf1-GFP with LD oil red staining (red). Localization of WT Atf1-GFP around stained LDs indicates LD localization. Quantification of fluorescence microscopy was performed by counting a minimum of 300 cells from three independent experiments. LD localization was observed in 91±1% of cells expressing wild type Atf1 and 0% for Δ2–48, Δ503–525, and the GFP control. In both oleic acid and glucose media WT Atf1-GFP associates with LDs. N- or C-terminal truncations (Δ2–48 and Δ503–525, respectively) result in cytosolic GFP signal indicating a loss of LD localization. Scale bar, 1 µm.

Deletions to the N- and C-termini of Atf1 were made to investigate the possible role of each terminus in membrane association (Figure 1A). Wild type Atf1, and Δ2–48 and Δ503–525 truncates tagged with GFP were constitutively overexpressed from a high copy number plasmid under the control of a PGK1 promoter. Cells were co-transformed with a single copy number plasmid expressing the LD marker Erg6. Fluorescent microscopy images of cells overexpressing Atf1-GFP and Erg6-DsRed cultured in oleic acid medium revealed co-localization of GFP and DsRed fluorescence, indicating that Atf1 localized to LDs (Figure 1B). Atf1-GFP also localized to LDs at stationary phase when expression was driven by a single copy plasmid (Figure S1). Deletions of residues 2–48 and 503–525 disrupted LD targeting resulting in dispersed Atf1-GFP fluorescence in the cytosol. These results were further substantiated by culturing cells in glucose medium. Fluorescent imaging of Atf1-GFP showed GFP fluorescence surrounding oil red stained LDs (91±1% of cells; Figure 1C). Corresponding to the results from the oleic acid cultures, the N- and C-terminal deletions resulted in cytosolic localization (0% LD localization, i.e. localization was not detected for Δ2–48, Δ503–525, and GFP control), thus confirming the importance of these two regions in membrane association.

### The predicted N-terminal amphipathic helix is required for membrane association of Atf1

**Figure 2:**
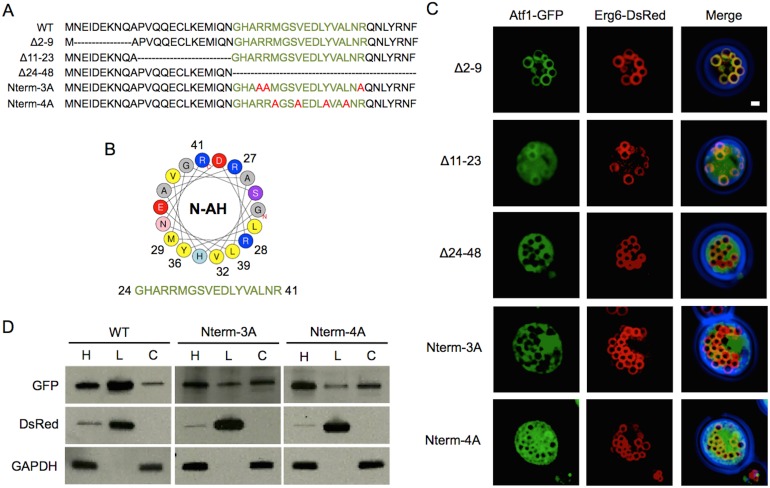
The N-terminal amphipathic helix of Atf1 is necessary for LD localization. (A) N-terminal sequences studied. The amino acid sequences of the predicted amphipathic helix are shown in green with point mutations to alanine shown in red. (B) Helical wheel projection of the N-terminal helix. Hydrophobic residues are colored yellow, neutral in gray, basic in blue, and acidic in red. The numbered basic residues were mutated to alanine in Nterm-3A and the numbered hydrophobic residues were mutated to alanine in Nterm-4A. (C) Fluorescent images of stationary phase cells cultured in oleic acid media overexpressing Atf1-GFP, truncates, and mutants with co-expression of the LD marker Erg6-DsRed. Truncation of residues 2–9 has no effect on LD localization while deletion of 11–23 and 24–48 attenuate LD localization. Mutation of residues in Nterm-4A and Nterm3A also attenuate LD localization. Scale bar, 1 µm. Quantification of fluorescence microscopy was performed by counting a minimum of 300 cells from three independent experiments. LD localization was observed in 89±4% of Δ2–9 expressing cells and 25±8%, 0%, 0%, and 4±1% of cell expressing Δ11–23, Δ24–48, Nterm-3A, and Nterm-4A, respectively. (D) Western blot analysis of cell homogenates (H), and LD (L) and cytosolic (C) cell fractions. Erg6-DsRed was used as a LD marker and GAPDH was used as a cytosolic protein marker.

To examine the possible molecular mechanism of Atf1 LD and the ER association, we predicted the secondary structure of the N-terminus [37]. The results strongly suggested that residues 11–48 form an alpha-helix. Analysis of this region by helical wheel projection [38] demonstrated that residues 24–48 were predicted to be an amphipathic helix (AH) (Figure 2A, B). The hydrophobic patch comprising Met29, Val32, Leu35, Tyr36, and Leu39 resides on one side of the helix, while polar residues Arg27, Arg41, Glu33, and Asp34 reside on the opposite side. To investigate the importance of the predicted helical structure, deletions including or excluding the predicted helix and point mutations on the AH were made. Deletion of residues 2–9 resulted in localization similar to the wild type, co-localization with Erg6 (89±4% LD localization; Figure 2C). Deletion of N-terminal 11–23 attenuated protein localization with a fraction of the Δ11–23 Atf1-GFP mutant dispersed in cytosol and some co-localized on LDs (25±8% LD localization). Deleting residues 24–48 of the N-terminal AH resulted in Atf1-GFP fluorescence accumulating in the cytosol (0% LD localization). These results infer that the AH structure has an important role on LD localization.

To confirm that LD association of Atf1 depends on the amphipathic character of the N-terminal helix (residues 24–48), point mutations were made to disrupt the helix. Basic residues Arg27, Arg28, and Arg41 were all mutated to alanine (Nterm-3A-GFP), resulting in cytosolic localization (0% LD localization). Hydrophobic residues Met29, Val32, Tyr36, and Leu39 were mutated to alanine (Nterm-4A-GFP), resulting partial LD localization (4±1% LD localization; Figure 2C). These results were confirmed by subcellular fractionation (Figure 2D). The majority of wild type Atf1-GFP was detected in LD fraction; however, a small fraction of the protein was also detected in cytosolic fraction, indicating that the cellular localization of Atf1 is not exclusively to LDs. AH disruption in Nterm-3A-GFP resulted a significantly reduced LD fraction and increased cytosolic fraction. Comparatively, Nterm-4A-GFP resulted in a reduction, but not complete elimination, of LD localization. Combined, these results suggest that the N-terminal AH is necessary but not sufficient for complete Atf1 LD association.

### The predicted C-terminal amphipathic helix is required for membrane association of Atf1

**Figure 3:**
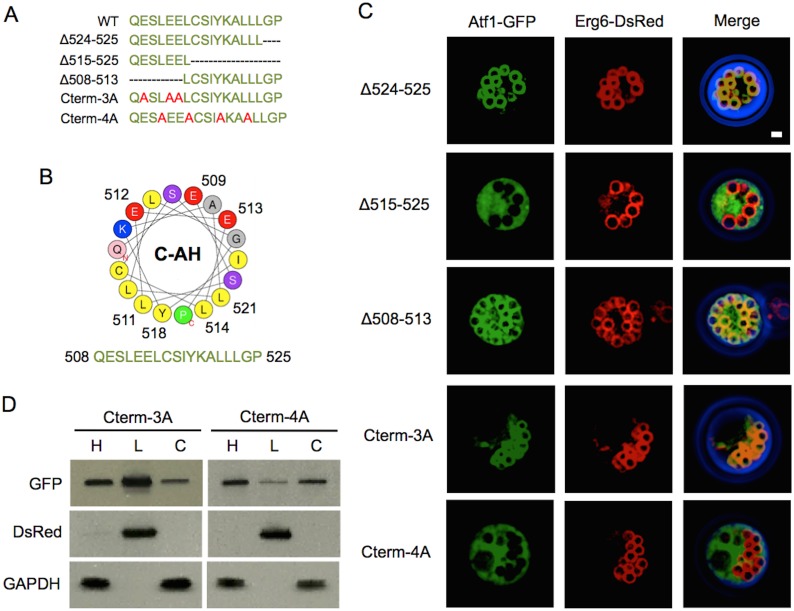
The C-terminal amphipathic helix of Atf1 is necessary for LD localization. (A) C-terminal sequences studied. Mutated residues shown in red and the amphipathic sequence shown in green. (B) Helical wheel projection of the C-terminal helix. Hydrophobic residues are colored yellow, neutral in gray, basic in blue, and acidic in red. The numbered basic residues were mutated to alanine in Nterm-3A and the numbered hydrophobic residues were mutated to alanine in Nterm-4A. (C) Fluorescent images of stationary phase cells overexpressing Atf1-GFP, truncates, and mutants with co-expression of the LD marker Erg6-DsRed. Truncation of residues 524–525 has no effect on LD localization while truncation of 515–525 and deletion of 508–513 eliminate LD localization. Mutation of residues in Nterm-4A attenuates LD localization, while Cterm-3A has little effect on LD localization. Scale bar, 1 µm. Quantification of fluorescence microscopy was performed by counting a minimum of 300 cells from three independent experiments. LD localization was observed in 89±1% of Δ524–525 expressing cells, while cells expressing Δ515–525, Δ508–513, Cterm-3A, and Cterm-4A showed LD localizations of 0%, 8±2%, 84±4%, and 0%, respectively. (D) Western blot analysis of cell homogenates (H), and LD (L) and cytosolic (C) cell fractions. Erg6-DsRed was used as a LD marker and GAPDH was used as a cytosolic protein marker.

Similar to the analysis of the N-terminal sequence of Atf1, C-terminal residues 503–525 were analyzed for secondary structure. Residues 508–525 were predicted as an AH (Figure 3A, B). The hydrophobic residues Leu511, Leu514, Cys515, Tyr518, Leu521, and Leu522 reside on one side of the helical wheel, while polar residues Lys519, Glu509, Glu512, and Glu513 reside on the opposite side. To investigate the significance of the AH at the C-terminus, deletions and mutations were made to key hydrophobic and acidic residues of the AH as well as to various parts of the C-terminus (Figure 3A). Deletion of the last two residues resulted in minimal change to LD association as judged by fluorescent microscopy (89±1% LD localization; Figure 3C). Deletion to residues 515–525, which includes the hydrophobic residues within the predicted AH, resulted in cytosolic localization (0% LD localization). In contrast, deletion of residues 508–513 could still localize the protein on LD (8±2% LD localization). These results suggest that the AH structure at the C-terminus serves an essential role in LD localization.

To confirm that the amphipathic property of C-terminal helix (residues 508–525) is important to membrane association, point mutations were made to eliminate the amphipathic character. The acidic residues Glu509, Glu512, and Glu513 were mutated to alanine (Cterm-3A-GFP). Surprisingly, the mutated proteins were still able to localize to LDs (84±4% LD localization). The discrepancy between deletion of 508–513 and mutations to the acidic residues of C-terminal AH may be due to a rotation of the helix resulting from the deletions. We next mutated four hydrophobic residues Leu511, Leu514, Tyr518, and Leu512 into alanine (Cterm-4A-GFP), resulting in a loss of membrane association (0% LD localization; Figure 3C). To verify the results of Cterm-3A-GFP and Cterm-4A-GFP subcellular fractionation and analysis of the fractions by western blot were performed (Figure 3D). Cterm-3A-GFP was found in LD and cytosolic fractions in similar ratios to wild type Atf1-GFP, most protein were distributed in LD fractions and only a small amount of proteins was localized in cytosolic fraction indicating that the acidic residues were not essential for LD localization. In contrast to Cterm-3A-GFP, LD localization of Cterm-4A-GFP was strongly reduced.

### N- and C-terminal amphipathic helices are required for membrane association of Atf2

**Figure 4:**
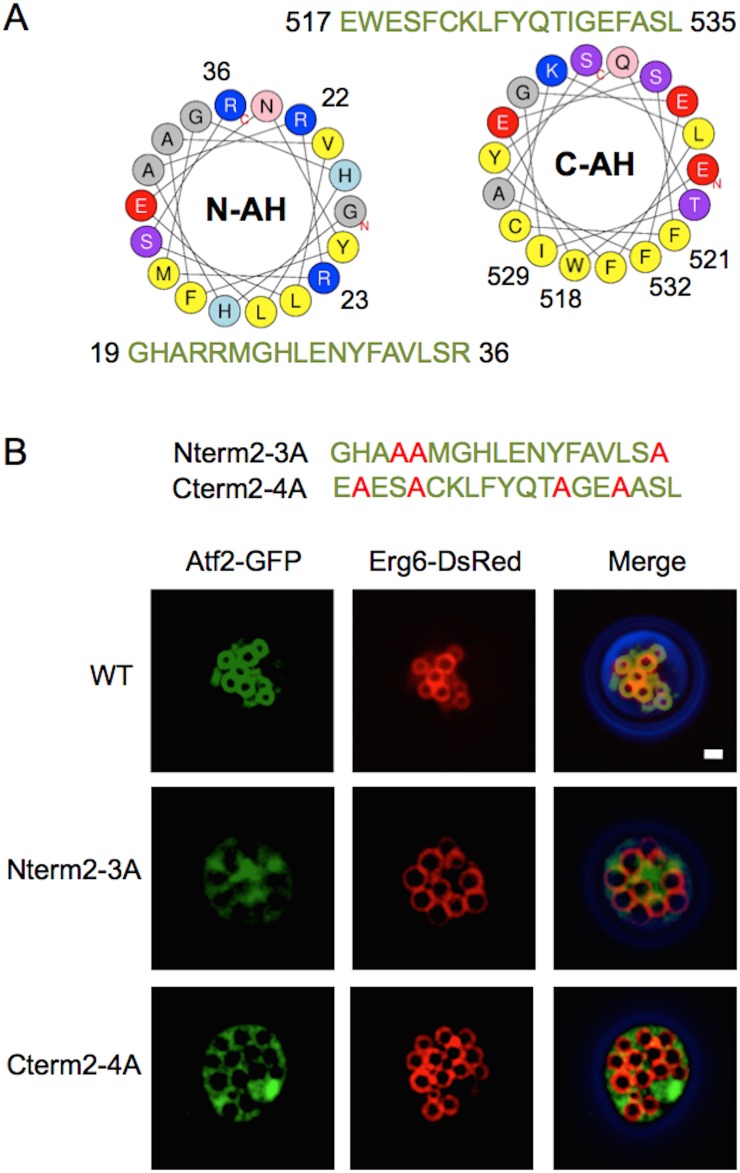
Atf2 from *S. cerevisiae* localizes to LDs. (A) Helical wheel projections of the N- and C-terminal domains of Atf2. Hydrophobic residues are colored yellow, neutral in gray, basic in blue, and acidic in red. The numbered basic residues were mutated to alanine in Nterm2–3A and the numbered hydrophobic residues were mutated to alanine in Cterm2–4A. (B) Fluorescent images of stationary phase cells overexpressing Atf2-GFP and mutants with co-expression of the LD marker Erg6-DsRed. Mutation of the basic residues in the N-terminal amphipathic helix (Nterm2–3A) and the hydrophobic residues in the C-terminal amphipathic helix (Cterm2–4A) eliminates LD localization. Scale bar, 1 µm. Quantification of fluorescence microscopy was performed by counting a minimum of 300 cells from three independent experiments. LD localization was observed in 80±3%, 0%, and 0% of cells expressing Atf2-GFP, Nterm2–3A, and Cterm2–4A, respectively.

To determine whether the structural homolog Atf2 can localize to LDs, we analyzed the sequence of Atf2 in a similar manner as Atf1. Both the N- and C-termini of Atf2 were predicted as AHs (Figure 4A) and Atf2-GFP was able to localize to LD (80±3% LD localization; Figure 4B). The similarity in terminal sequences and LD localization suggested that Atf2 might share the same LD localization mechanism as Atf1. To explore this possibility, polar residues Arg22, Arg23, and Arg36 were mutated into alanine on the predicted N-terminal AH and the hydrophobic residues, Trp518, Phe521, Ile529, and Phe532 were mutated to alanine on the predicted C-terminal AH. Disruption to both the N- and C-terminal AHs resulted in a loss of LD localization as both Atf2-GFP mutants were found to be expressed in the cytoplasm (0% LD localization; Figure 4B). These results suggested that the targeting mechanism of LD localization of Atf1 and Atf2 are conserved in *S. cerevisiae*.

To rule out the possibility that mutations to Atf2 produced mis-folded or truncated protein, we analyzed expression by western blot. There was no apparent degradation of overexpressed Atf2 or its mutants, suggesting that the fusions of Atf2 and GFP are well-folded and not susceptible to proteolysis (Figure S2). Similar results were found with wild type and mutant Atf1. In addition, fluorescence images of Atf1, −2 and their corresponding mutants did not exhibit significant aggregation, suggesting that fusion proteins are well folded.

### N- and C-terminal amphipathic helices of Atf1 and −2 are necessary for ER association prior to LD sorting

**Figure 5:**
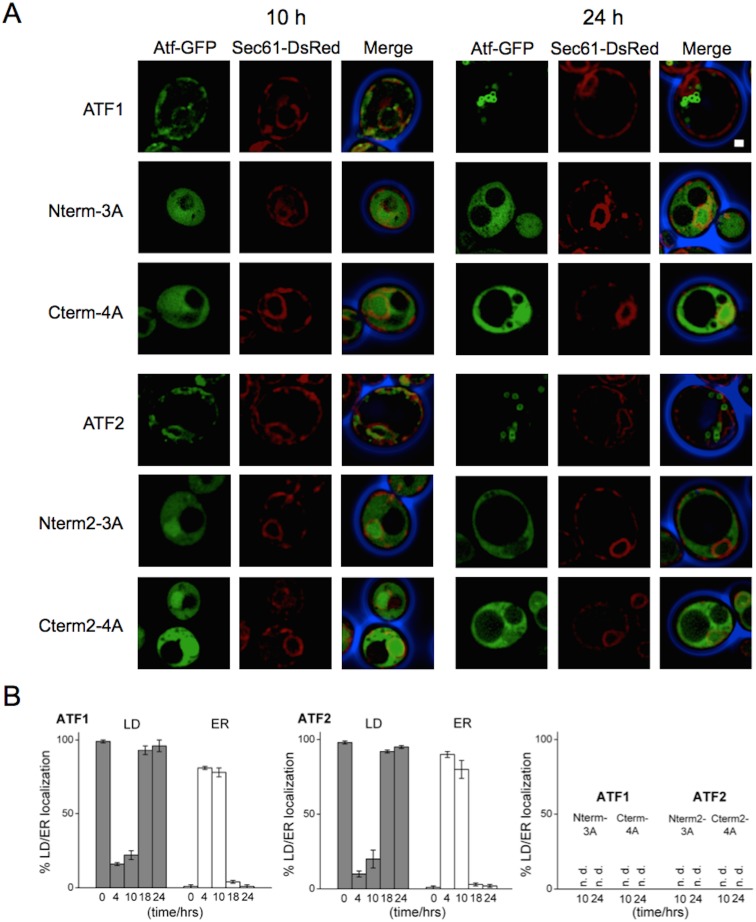
Atf1 and Atf2 from *S. cerevisiae* localize to LDs via the ER. (A) Cells overexpressing wild type and mutant Atf1 and −2 were cultured in synthetic uracil dropout media with 2% glucose to log phase and stationary phase and analyzed by fluorescence microscopy. The ER is identified by chromosomal tagging of the ER marker Sec61 with DsRed. Wild type Atf1 and −2 associate with the ER during log phase and preferentially sort to LDs during stationary phase. Disruption of the N- or C-terminal amphipathic helices in both Atf1 and −2 prevents ER association and subsequent localization to LDs. Scale bar, 1 µm. (B) Quantification of LD and ER localization at different time points. Data are plotted as mean±SD. A minimum of 300 cells were counted from three independent experiments.

Our results thus far (Figures 1–4) demonstrate the necessity of terminal AH domains for LD targeting of Atf1- and −2, but they do not indicate a trafficking mechanism. Figure 5 shows a time course study of the intracellular localization of Atf1 and −2 at time points of 0, 4, 10, 18, and 24 hours. For the initial time point (0 hours), cells were grown to stationary phase prior to dilution in sterile media. In lag and early log phases (4 and 10 hours, respectively) ∼80% GFP tagged Atf1 and −2 co-localized with the DsRed tagged ER marker Sec61 (Figure 5B). Atf1 and −2 were observed to shift from the ER to LDs as cell progressed from late log phase to stationary phase, suggesting that the transport is growth dependent (Figure 5B and Figure S3). In addition, the formation of nascent and premature LDs observed by GFP tagged Atf1 and −2 suggests the process is ER associated, consistent with current models of LD formation [22]–[24]. Disruption of either terminal AH in Atf1 and −2 (Nterm-3A, Cterm-4A, Nterm2–3A, and Cterm2–4A, respectively) prevented association with the ER in log phase, thus resulting in localization to the cytosol. Localization to the ER or LDs is not recovered in stationary phase, suggesting that the inability of the mutants to localize to LDs is a consequence of disrupted ER targeting.

### Terminal AH domains of Atf1 are necessary but not sufficient for ER and LD localization

**Figure 6:**
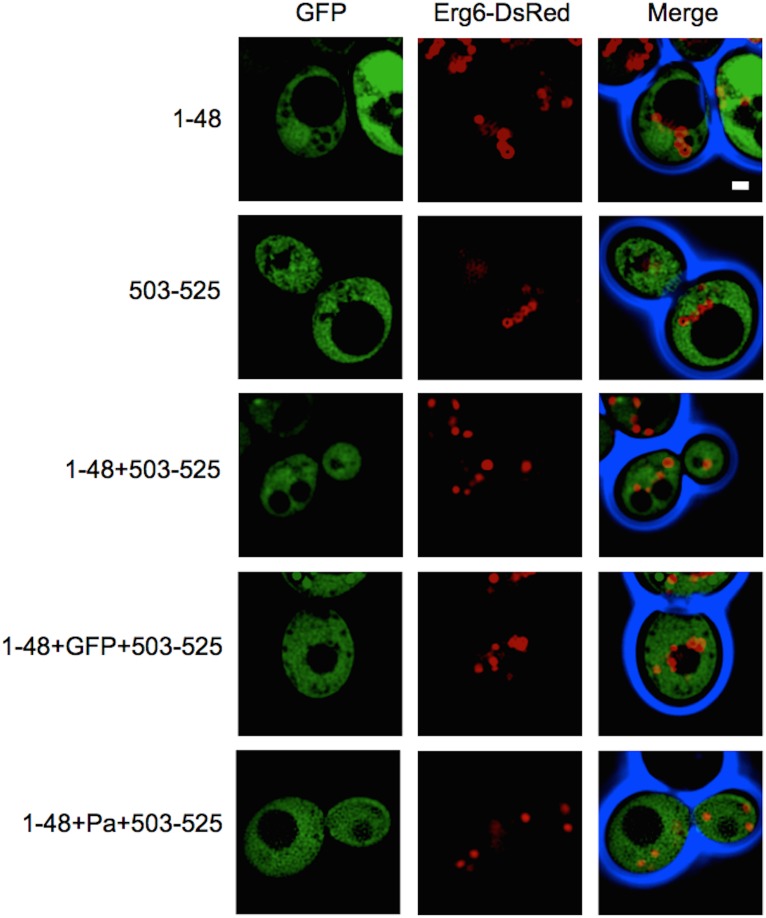
The N- and C-terminal amphipathic helices of Atf1 are not sufficient to localize GFP to LDs. Fluorescence images of stationary phase cells overexpressing Atf1 amphipathic helix-GFP fusions, including N-terminal (1–48), C-terminal (503–525), both N- and C-terminal helices fused to the N-terminus of GFP, and 1–48 fused to the N-terminus of GFP with 503–525 fused to the C-terminus of GFP. Fusion of N- and C-terminal helices of Atf1 (*S. cerevisiae*) to Atf from *P. anomala* is also shown. Lipid droplets are identified with chromosomally tagged Erg6-DsRed. Scale bar, 1 µm. Quantification of fluorescence microscopy was performed by counting a minimum of 300 cells from three independent experiments. No significant localization to LDs was observed (0% for all protein constructs).

While the N- and C-terminal AHs of Atf1 are responsible for ER targeting and eventual sorting to LDs, fusion of one or both helices to GFP fails to produce the same function (Figure 6). N-terminal fusion of residues 1–48 and, separately, C-terminal fusion of residues 503–525 to GFP results in cytosolic expression. Similarly, N-terminal fusion of both helices (1–48 and 503–525) and N- and C-terminal fusions of 1–48 and 503–525, respectively, resulted in cytosolic expression. All fusions of one or more terminal AH domains from Atf1 to GFP were dispersed in cytosol, thus indicating that these fragments are not sufficient for ER and LD targeting. These results were reproduced with a second reporter, GFP tagged Atf from *Pichia anomala*. N- and C-terminal fusions to Atf-Pa (1–48+Atf-Pa+503–525) with C-terminally fused GFP resulted in cytosolic protein expression and no LD localization was observed.

### N- and C-terminal amphipathic helices are essential for membrane association of Atfs in *S. cerevisiae*

**Figure 7:**
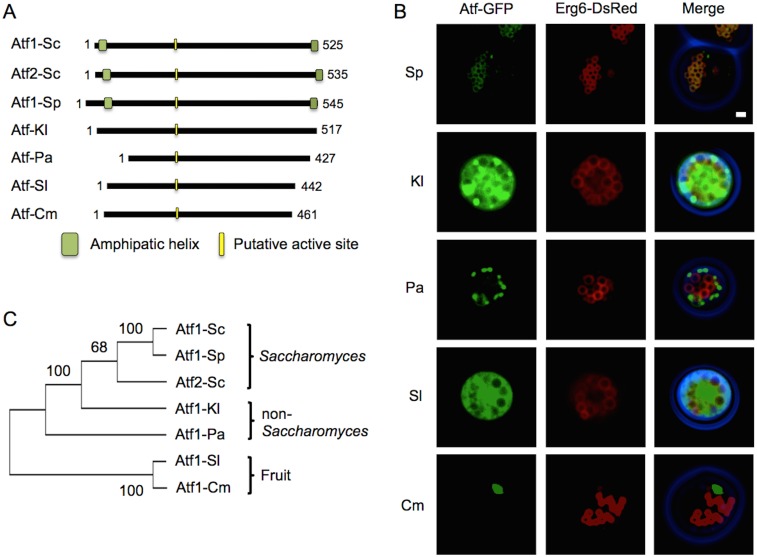
N- and C-terminal amphipathic helices are necessary for LD localization of Atfs. (A) Schematic representation of the amino acid sequences of Atf orthologs from *S. cerevisiae* (Sc) *S. pastorianus* (Sp), the non-Saccharomyces yeast *Kluyveromyces lactis* (Kl) and *Pichia anomala* (Pa), and the fruit *Solanum lycopersicum* (Sl) and *Cucumis melo* (Cm). Predicted amphipathic helices and the putative active site (HXXXD) are shown in green and yellow, respectively. The length of the primary sequence is given at the end of the schematic for each enzyme. (B) Fluorescent images of stationary phase cells co-expressing Atf-GFP and the LD marker Erg6-DsRed. Atf1 from the *Saccharomyces* yeast *S*. *pastorianus* localizes to LDs, while orthologs that do not have predicted amphipathic helices at the N- and C-termini, including *K. lactis*, *P. anomala*, *S. lycopersicum*, and *C. melo* do not localize to LDs. Scale bar, 1 µm. (C) Phylogenetic tree of the studied Atfs. Amino acid sequences were obtained from National Center for Biotechnology Information and aligned by Clustal W [57]. Phylogenetic analysis was implemented in Mega 5.2.2, and trees were constructed by neighbor-joining algorithm [58]. Bootstrap values are displayed at the nodes.

Several AATases from yeast and fruit species have been identified and enzymatic activity towards short linear and branched chain alcohols has been demonstrated. A bioinformatic analysis comparing fungal orthologs of Atf1 and Atf2 shows a conserved putative active site, H-X-X-X-D (Figure 7A) [14]. Using the active site as a common point of alignment, the sequences of Atf orthologs from *Saccharomyces pastorianus*, the non-*Saccharomyces* yeasts *Kluyveromyces lactis* and *Pichia anomala,* and the fruit species *Solanum lycopersicum* and *Cucumis melo* were compared with Atf1 and −2. From the alignment it was apparent that Atf orthologs differ, among other things, in total length (by as many as 98 amino acids) and differ in the length of the terminal domains. Atf1 from *S. pastorianus* (Atf1-Sp) is 545 amino acids in length and is predicted to have conserved amphipathic structures at the termini. Atf from *K. lactis* (Atf-Kl) is similar in length to Atf1 from *S. cerevisiae* but the predicted terminal amphipathic helices are missing. Atfs from *P. anomala*, *S. lycopersicum*, and *C. melo* (Atf-Pa, -Sl, and -Cm, respectively) are shorter in length and also do not have predicted AHs at the termini. To examine whether the presence of terminal AHs can localize enzymes to LD, we heterologously expressed these Atfs in *S. cerevisiae*. Similar to Atf1-GFP and Atf2-GFP from *S. cerevisiae*, Atf1-Sp-GFP co-localized with Erg6-DsRed (Figure 7B). In contrast, the absence of terminal AHs disabled enzyme localization to LDs: Atf-Kl-GFP was unevenly distributed in cytoplasm; Atf-Pa-GFP tended to form punctates in cytoplasm; Atf-Sl-GFP was evenly dispersed in cytoplasm; and, Atf-Cm-GFP was prone to aggregate in cytoplasm. These results support the notion that terminal AHs are important for Atf LD localization *S. cerevisiae*.

To understand how these AHs evolved, a phylogenetic tree was constructed (Figure 7C). The selected Atfs segregated into two distinct evolutionary groups–yeast and fruits. The terminal AHs appeared to have developed along the *Saccharomyces* branch of the yeast Atfs. While similar helices and the corresponding function of LD localization did not develop in non*-Saccharomyces* yeast or in fruit species.

## Discussion

Atf1 and Atf2 from *S. cerevisiae* have been the focus of considerable research from fermented beverage industries and from metabolic engineers. Brewers and wine makers are interested in Atf activity and its role in creating aromas and flavors in their products [4], [7]–[9], while metabolic engineers have goals of industrial production of short chain esters [39]–[43]. The biochemistry of Atf2 has also been investigated for its ability to produce sterol esters [20] and its role in the export of sterols and steroids [21]. Here, we explored the intracellular localization of these enzymes and formulate a potential mechanism for such spatial organization. We demonstrated that membrane association of Atf1 requires dual amphipathic helices at the enzyme’s termini (Figures 1, 2 and 3). The necessity of the N- and C-terminal amphipathic helices for membrane association is also demonstrated in the Atf2 homolog (Figure 4). In both cases, disruption of the terminal helices prevents association with the ER, thereby eliminating LD localization (Figure 5). Finally, a comparison of seven Atfs from yeasts and fruits revealed this mechanism of LD localization is common to *S. pastorianus* and that only those Atfs with dual amphipathic helices localize to LDs (Figure 7).

Deletion studies reveal that N-terminal residues 2–48 and C-terminal residues 503–525 of Atf1 are necessary for LD localization (Figure 1). We predicted that sections of these terminal sequences form AHs and our data demonstrates their involvement in LD and ER membrane association. The absence of multiple proline residues within these sequences suggests that it is highly unlikely that either termini form hairpin loops, a motif that is known to mediate membrane association [44]. In addition, it is unlikely that Atf1 associates with LD or ER membranes via a lipid anchor. It has been reported that Rab18 may target LDs with a prenylated lipid anchor conjugated to a CaaX motif at the C-terminus [45], [46], where C is cysteine, “a” are aliphatic residues, and X is any amino acid. Sequence analysis of C-terminal AH revealed no such motif in Atf1. The possibility that a protein mediates membrane association remains to be investigated; however, our deletions and point mutations to Atf1 that remove or disrupt AHs at the termini suggest a mechanism of AH to membrane association (Figures 1–5). Fusions of Atf1 AHs to GFP and Atf-Pa are not able to localize the fluorescent protein to LDs or the ER (Figure 6); therefore, we proposed that the terminal AHs of Atf1 are necessary but not sufficient for ER dependent, LD localization.

AH binding to phospholipid membranes depends on interactions between hydrophobic residues and the lipid membrane as well as electrostatic interactions between basic residues and negatively-charged phosphate groups of the phospholipid layer [47]. Our results show that amphipathic character of the N- and C-termini of Atf1 and −2 are necessary for LD localization and association with the ER. Mutation of the hydrophobic residues of each AH disrupts localization (Figure 2, 3, 4 and 5). These results are consistent with previously identified AH-dependant LD proteins including the antiviral protein, viperin, where reduced localization was observed with mutation to the hydrophobic residues of an N-terminal AH [48]. Similar to our results with the C-terminal AH in Atf1, mutations to acidic residues in the viperin AH did not influence membrane binding, indicating that the acidic residues are dispensable (Figure 3C).

One significant difference between Atf1, Atf2, viperin and other LD proteins that associate by an AH mechanism is the necessity of dual AH domains. In many cases the identified LD localizing AH is sufficient to target GFP to LDs [48]–[50]. In the case of Atf1 and −2, disruption of one of the two helices attenuates localization. Similar type behavior is observed in a Hepatitis C virus (HCV) core protein where two AHs connected by a hydrophobic loop are needed for LD localization [51]. Moreover, fusions of Atf1 AHs to GFP do not localize to the ER or LDs, leading to the conclusion that N- and C-terminal AHs of Atf1 are necessary but not sufficient for LD targeting. A similar conclusion has been drawn for murine diacylglycerol acyltransferase-2 (DGAT2), a transmembrane protein transiently localized to LDs [52]. Two putative LD localization domain, a proline knot motif and an 18 amino acid hydrophobic region, are necessary for sorting from the ER to LDs, but fusion to a fluorescent protein demonstrates that the domains are not sufficient for LD localization. In the case of Atf1, it is possible that one or more hydrophobic patches within the protein may also be involved with ER association and subsequent sorting to LDs.

It has previously been reported that Atf2 localizes to the ER during exponential growth [21]. It has also been reported that 85% of Atf2 activity from *S. cerevisiae* purifies from lysates with cell fractions found in the supernatant of 25,000 rpm centrifugations [20]. While it was not explicitly shown, these fractions likely contain LDs [53], [54]. These two reports and our data demonstrating Atf2 LD localization are mutually supportive. The prevailing model of LD genesis is one of LDs budding from the ER with LD proteins originating on the cytosolic side of the ER [23]. Both Atf1 and −2 exhibit this behavior, associating with the ER during exponential phase growth sorting to LDs as they bud from the ER in stationary phase (Figure 5).

In reporting Atf2 localization to the ER, Tiwari et al. [21] showed that the N- and C-termini of Atf2 were resistant to proteinase K digestion, suggesting that these two regions are protected in the ER lumen. Our results show that the terminal AHs are necessary for the membrane association of Atf2 and −1 through a putative mechanism of AHs binding to the ER and LDs (Figure 5). This is consistent with the proteinase protection result as the AH domains are partially embedded in the membrane and protected from proteinase activity.

Our data and a previous report [18] clearly demonstrate Atf1 LD localization, and for the first time we demonstrate that Atf2 also localizes to LDs in *S. cerevisiae*. The biological function of this localization remains unclear. One possible explanation for Atf2 localization is its activity towards acetylation of sterols [20], [21]. Steryl ester hydrolases that release sterols from LDs also localize the LD membrane [55], thus presenting the possibility of transient metabolon formation between Atf2 and steryl ester hydrolases. In such a case, released sterols would be locally accessible for acetylation by Atf2. It is also possible that transient metabolons form with Atf1. It has been suggested that biological role of Atf1 is the regeneration CoA without concomitant production of toxic acids [4], [56]. The regenerated CoA would then be locally accessible to LD localized acyl-CoA synthetases that activate free fatty acids mobilized from LD TAGs [25]. Studies exploring possible protein-protein interactions between Atf2 and LD bound hydrolases or Atf1 and LD bound acyl-CoA synthetases have not yet been conducted and represent an interesting new avenue of research.

## Supporting Information

Figure S1 **Fluorescence microscopy analysis of cellular localization of Atf1p under low expression conditions.** Protein expression was driven by PGK1 promoter in the single copy number plasmid CEN/ARS. Erg6 is the lipid droplet marker and the fluorescence signal is from DsRed tagged to C-terminal of Erg6 on the chromosome. The cells were grown on 2% glucose and cultured to stationary phase. Scale bar 1 µm.
(DOCX)

Figure S2 **Western blot analysis of Atf1, −2 and their mutants.** All enzymes are C-terminally tagged with GFP.
(DOCX)

Figure S3 **Time course study of cellular localization of Atf1, Atf2, and their mutants.** Cells were first grown to stationary phase followed by dilution in fresh media (t = 0 hrs.). Cells were harvested and analyzed at different time points (t = 4, 10, 18, and 24 hrs.). Nascent and premature LDs are indicated by white arrows.
(DOCX)
