# Supplementary figures and images for: Dual N- and C-Terminal Helices Are Required for Endoplasmic Reticulum and Lipid Droplet Association of Alcohol Acetyltransferases in Saccharomyces cerevisiae
Source: PLoS One. 2014 Aug 5;9(8):e104141. doi: 10.1371/journal.pone.0104141 (PMC4122449; doi:10.1371/journal.pone.0104141)

Figure S1


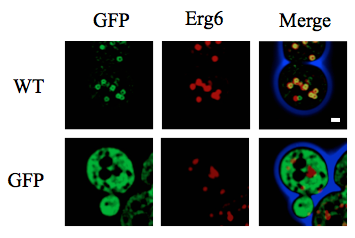

Supplement: Figure S1 — Fluorescence microscopy analysis of cellular localization of Atf1p under low expression conditions. Protein expression was driven by PGK1 promoter in the single copy number plasmid CEN/ARS. Erg6 is the lipid droplet marker and the fluorescence signal is from DsRed tagged to C-terminal of Erg6 on the chromosome. The cells were grown on 2% glucose and cultured to stationary phase. Scale bar 1 µm. (DOCX) [file pone.0104141.s001.docx]

Figure S2


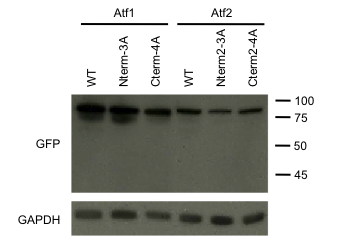

Supplement: Figure S2 — Western blot analysis of Atf1, −2 and their mutants. All enzymes are C-terminally tagged with GFP. (DOCX) [file pone.0104141.s002.docx]

Figure S3


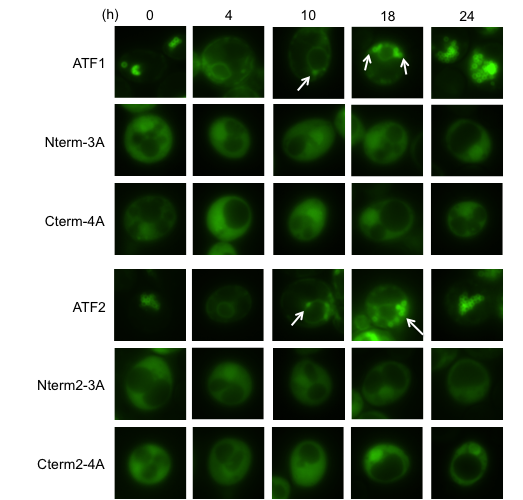

Supplement: Figure S3 — Time course study of cellular localization of Atf1, Atf2, and their mutants. Cells were first grown to stationary phase followed by dilution in fresh media (t = 0 hrs.). Cells were harvested and analyzed at different time points (t = 4, 10, 18, and 24 hrs.). Nascent and premature LDs are indicated by white arrows. (DOCX) [file pone.0104141.s003.docx]
